# Exploring the Upper Limits of Cerebral Perfusion Pressure in Pediatric Traumatic Brain Injury: A STARSHIP Analysis

**DOI:** 10.1007/s12028-025-02358-2

**Published:** 2025-09-08

**Authors:** Stefan Yu Bögli, Ihsane Olakorede, Claudia Ann Smith, Peter Hutchinson, Marek Czosnyka, Peter Smielewski, Shruti Agrawal, The STARSHIP Study Team

**Affiliations:** 1 Brain Physics Laboratory, Division of Neurosurgery, Department of Clinical Neurosciences, University of Cambridge, Cambridge, UK; 2 Department for Neurology and Neurocritical Care Unit, Neuroscience Center Zuricht, University of Zurich and University Hospital Zurich, Zurich, Switzerland; 3 Division of Neurosurgery, Department of Clinical Neurosciences, University of Cambridge, Cambridge, UK; 4 Institute of Electronic Systems, Warsaw University of Technology, Warsaw, Poland; 5 Department of Paediatrics, Cambridge University, Cambridge, UK; 6 Paediatric Intensive Care, Cambridge University Hospitals, Cambridge, UK

**Keywords:** Pediatric traumatic brain injury, Cerebral perfusion pressure, Cerebrovascular autoregulation, Multimodality monitoring

## Abstract

**Background:**

Low cerebral perfusion pressure (CPP) has previously been identified as a key prognostic marker after pediatric traumatic brain injury (TBI). Cerebrovascular autoregulation supports stabilization of cerebral blood flow within the autoregulation range. Beyond the upper limit of this range, cerebral blood flow increases with increasing CPP, leading to increased risk of intracranial hypertension and blood–brain barrier disruptions. Based on the hypothesis that children are less sensitive to high CPP, we aimed to characterize the pediatric upper limit of autoregulation and the association between high CPP and outcome.

**Methods:**

Data acquired as part of the "Studying Trends of Autoregulation in Severe Head Injury in Paediatrics" (STARSHIP) study (a prospective, multicenter, observational study that enrolled 135 children with TBI from July 2018 to March 2023) were explored. The association between different levels of CPP and the autoregulation proxy measure, the pressure reactivity index (PRx), were explored visually. The prognostic value of CPP was assessed by exploring overall averages, overall dose, hourly dose, and percentage time spent above specific thresholds. We employed univariable/multivariable (χ^2^ tests, logistic regression, sliding dichotomy) and visual (heatmap) methods.

**Results:**

No clear upper limit of autoregulation could be identified with PRx increasing beyond 0.2 only with CPP values beyond 100 mm Hg. Using iterative χ^2^ testing and logistic regression analyses, similarly, only hourly dose and percentage time beyond CPP of 90 mm Hg displayed a trend toward worse outcome. Using heatmap analyses, regions of CPP with differing risk stratifications could be identified. No difference in CPP could be identified between patients with and without acute respiratory distress syndrome or secondary hemorrhages.

**Conclusions:**

In contrast to the well-established association between low CPP and poor outcome, our findings suggest that exposure to CPP values above those recommended by the Brain Trauma Foundation guidelines may not be associated with worse outcomes in this cohort. However, given the observational nature of the study and potential confounding factors, these results highlight the need for prospective trials to assess the safety and efficacy of targeting higher CPP in pediatric TBI.

**Supplementary Information:**

The online version contains supplementary material available at 10.1007/s12028-025-02358-2.

## Introduction

Cerebral perfusion pressure (CPP) is widely regarded as a key target for neurocritical care management following pediatric traumatic brain injury (TBI) [1, 2]. CPP quantifies the driving pressure (i.e., difference between arterial blood pressure [ABP] and intracranial pressure [ICP]) of cerebral blood flow (CBF). Cerebrovascular autoregulation helps to maintain a stable CPP by adjusting arteriolar diameters in response to changes in ABP and thus protects the brain from large fluctuations in CBF [3]. It functions optimally within a defined range bound by the lower and upper limits of autoregulation (LLA and ULA, respectively). At the bedside, the pressure reactivity index (PRx) is often used as a surrogate measure for quantification of cerebrovascular autoregulation [4]. Below the LLA, CBF decreases linearly with declining CPP, which increases the risk of hypoxic injury [5]. Substantial evidence has demonstrated the relevance of sustaining CPP above the LLA for prognostication [6, 7]. Conversely, exceeding the ULA may promote secondary brain injury by causing a linear rise in CBF and hyperperfusion, potentially elevating ICP and inducing hyperemic edema. Although adult guidelines advise caution against aggressive maintenance of CPP above 70 mm Hg to avoid complications such as respiratory failure [8, 9], robust evidence to establish safe upper limits remains limited in adult populations and is virtually absent in pediatric cohorts. At the same time, available data hint at a relatively large autoregulatory plateau in the pediatric cohort [10].

Based on previous findings that identified the LLA as the most promising but dynamically changing prognostic factor, it would be of utmost clinical importance to understand to what extent CPP can be raised beyond the LLA. Based on the hypothesis that children exhibit a greater tolerance to elevated CPP, this analysis investigates the relationship between different CPP cutoffs and outcome in data acquired as part of the multicenter prospective study Studying Trends of Autoregulation in Severe Head Injury in Paediatrics (STARSHIP) [11].

## Materials and Methods

This study represents a secondary analysis of the STARSHIP study [11, 12], which was a multicenter prospective observational study assessing cerebrovascular reactivity in a cohort of 135 pediatric patients with TBI enrolled across ten centers within the UK. Nine of these centers are children’s major trauma centers, representing centers that provide specialized trauma care with an interdisciplinary team of physicians. The inclusion/exclusion flow chart has previously been described [11]. Of 165 screened patients, 135 were recruited for the mortality-based analysis and 124 were recruited for the outcome analyses. The median number of patients acquired per center was 4 (interquartile range [IQR] 2–9). The management of these patients across the different centers follows the pediatric Brain Trauma Foundation guidelines [1]. These guidelines currently do not include management based on PRx. Although the study laptops acquiring the data were at the bedside of the patients, treating clinicians were not trained to interpret or act upon these values, and PRx was not part of clinical protocols during the study period, rendering the likelihood that PRx directly influenced management decisions very low. STARSHIP was approved by the Health Research Authority, South West-Central Bristol Research Ethics Committee (ref: 18/SW/0053, 23/SW/0011). The study was conducted in accordance with the ethical principles set forth in the 1964 Declaration of Helsinki and its subsequent revisions. Informed consent was acquired prior to hospital discharge for follow-up from the patient’s parents/guardians.

The primary objective of this study was to characterize the ULA in pediatric patients with TBI and to assess whether exposure to elevated CPP is associated with 12-month outcomes. The primary hypothesis was that children exhibit greater physiological tolerance to elevated CPP and that high CPP values are consequently not associated with worse outcomes. Secondary objectives included examining the relationship between CPP across the full available range and PRx, as well as assessing the association between elevated CPP and secondary complications, such as acute respiratory distress syndrome and secondary intracranial hemorrhage.

### Clinical Data

Clinical and demographic data were collected as previously described [11]. Outcome was evaluated at 12 months using the pediatric Glasgow Outcome Scale Extended (GOSE Peds) by trained personnel. The frequency of acute respiratory distress syndrome was assessed systematically based on consensus criteria [13]. The incidence of secondary hemorrhages—defined as those occurring after the initial imaging obtained upon hospital admission—was assessed using computed tomography scans performed at the discretion of the treating physicians. ICP monitoring was initiated based on established criteria largely aligned with the Brain Trauma Foundation guidelines [1].

### Multimodality Monitoring Data Acquisition, Curation, and Processing

Data acquisition, curation, and preprocessing were performed as previously described [11]. All patients received invasive ICP monitoring using intraparenchymal probes (Codman ICP MicroSensor, Codman & Shurtleff, Raynham, MA) and invasive ABP monitoring using a radial or femoral arterial line (Baxter Healthcare, Deerfield, IL) zeroed at the level of the right atrium. The waveform resolution data was acquired at a resolution of 250 Hz within bedside laptops using the ICM + software (Cambridge Enterprise, Cambridge, UK). Data curation was performed using the ICM + software and included manual removal of large artifacts representing nonphysiological disturbances (e.g., device disconnections or arterial line failure) and automated artifact markup of sections lacking a pulse or values outside physiological ranges. PRx was calculated as previously described [4], by computing the moving correlation coefficient between 10-s mean ABP and ICP values over consecutive 5-min intervals. Optimal CPP (CPPopt), LLA, and ULA were estimated automatically, as previously described, by fitting a parabolic curve to 5-min median CPP and PRx values [11, 14]. For the subsequent analysesQuery, minute-by-minute averages were used.

### Statistical Analysis and Visual Exploration

Statistical analyses and figure preparation were performed using R studio software (version 4.4.1 https://www.r-project.org/; the following packages were used: *dplyr*, *rstatix*, *gtsummary*, *MASS*, *logistf*, *ggplot2*). The primary analysis was performed with outcome dichotomized as favorable vs. unfavorable outcome (GOSE Peds 1–4 versus 5–8; available for 124 patients). The secondary analyses, including the sliding dichotomy approach and the heatmap exploration, explored the full ordinal GOSE Peds scale. A significance level of *p* < 0.05 was set with Bonferroni adjustments applied to control for multiple comparisons where appropriate.

The relationship between CPP and PRx was initially explored using visual methods. Specifically, for each patient, the median PRx within CPP bins (5-mm Hg intervals ranging from 30 to 120 mm Hg) was calculated, along with the number of data points in each bin. These were then aggregated and visualized using histograms and boxplots either across the entire dataset (to quantify the overall state of autoregulation) or stratified by outcome (favorable or unfavorable) or age group (0–2 vs. 2–8 vs. > 8 years; cutoffs chosen in line with previous research [10, 11]).

For statistical analysis, both deviations from the dynamically calculated ULA [11] and a series of fixed CPP targets ranging from 60 mm Hg (near the upper limit of the LLA identified in the prior analysis) to 120 mm Hg (in increments of 5 mm Hg) were explored. For each of these CPP targets, we calculated three metrics: (1) dose, defined as the area above the target yet below the actual CPP over the total monitoring period; (2) hourly dose (hDose), representing the dose normalized per hour of valid data; and (3) percentage time (ptime), quantifying the proportion of monitoring time during which CPP exceeded the target.

Two univariable methods were employed to extrapolate the association between different CPP cutoffs and outcome. First, iterative χ^2^ tests with Yates correction for small sample size were employed to assess the association between different CPP cutoffs and outcome. Second, iterative logistic regression models were used to assess the association and magnitude of associations between CPP dose, hDose, and ptime at different levels and outcome. To account for differences in clinical severity and due to the ordinal nature of the GOSE Peds scale, a sliding dichotomy approach was applied [11, 15]. Briefly, for each patient, we estimated a prognostic risk score (estimated using logistic regression including the variables age, motor GCS, pupillary reactivity, type of TBI [isolated vs. polytrauma], injury severity score, Rotterdam score, hypoxia, hypotension, cardiac arrest, ICP, and dose below the LLA), which was then used to create a relative outcome scale for each patient. Specifically, based on the prognostic risk scores, the patients were divided into three groups of roughly equal size with low, intermediate, and high likelihood of unfavorable outcome. The definition of favorable and unfavorable outcome was then adjusted depending on the prognostic risk score for each patient. In patients with low likelihood only, GOSE Peds of 1–2 was considered favorable, whereas for the patients with intermediate likelihood and high likelihood, GOSE Peds of 1–4 and GOSE Peds of 1–6, respectively, were considered favorable. Given the relatively small sample size and the potential for estimation bias due to the dichotomous outcome and the continuous predictors, we employed Firth’s penalized likelihood logistic regression. This method corrects for small sample bias and mitigates the problem of quasi-complete separation by introducing a bias-reducing penalty.

### Heatmap Exploration

To allow for deeper insight into the CPP dynamics, we explored visualizations based on the initial description by Güiza et al. [16]. For this purpose, a publicly available code [17] was used and adapted to fit the current analysis. Two heatmaps were produced. First, to explore the association between magnitude and duration of CPP relative to the LLA and outcome, minute-by-minute deviations of CPP from the LLA were explored. For each patient, a grid describing the frequencies of different combinations of minimum deviation (range − 30 to 60 mm Hg, 2 mm Hg per cell) from the LLA and minimum duration (range 5 to 120 min, 2 min per cell) were developed. Second, to explore the impact of different combinations of CPP and ICP levels, the minute-by-minute CPP and ICP data were explored to create a grid per patient describing the percentage monitoring time with specific combinations of CPP (range 50 to 110 mm Hg, 2 mm Hg per cell) and ICP (from 5 to 40 mm Hg, 2 mm Hg per cell). To create the heatmaps, these heatmaps were then combined, and for each cell, weighted Pearson correlation (using the number of patients as weights) was used to assess the relationship between the average frequency (for deviations of CPP from the LLA) and ptime (for the CPP/ICP relationship) per outcome category. The resulting maps were then visualized using a color map ranging from orange to green, representing associations with worse and better outcome, respectively. To improve visual interpretability, cells were smoothed, as previously described [18], whereby each cell of the grid was divided into 3 × 3 smaller cells, and this was followed by application of a Gaussian kernel filter (standard deviation of 2 pixels). Individual grid cells with data coming from less than 20 patients (representing roughly 15% of patients) were colored gray.

## Results

One hundred thirty-five children requiring invasive clinical monitoring for TBI were included in this analysis; the STARSHIP cohort has been previously described [11]. The clinical parameters are presented in Table 1. There was an average of 109 (IQR 76–170) hours of high-frequency physiological monitoring data per patient. The CPPopt (and correspondingly LLA and ULA) yield was 88%. The median ICP was 13.8 (IQR 11.6–16.4) mm Hg for patients with favorable outcome vs. 15.5 (IQR 13.7–18.7) mm Hg for patients with unfavorable outcome (*p* = 0.011).

**Table 1:** Patient characeristics

Characteristic	Entire cohort (*N* = 135)
Sex (male)	105 (78%)
Age (months)	96 (26–152)
*Type of injury*	
Blunt	130 (96%)
Penetrating	5 (3.7%)
*Isolated/polytrauma*	
Isolated	63 (47%)
Polytrauma	72 (53%)
*Mechanism of injury*	
Road traffic accident	63 (47%)
Fall	27 (20%)
Bicycle accident	24 (18%)
Other	11 (8.1%)
Inflicted injury	10 (7.4%)
Mortality	10 (7.4%)
Unfavorable outcome	44 (35.5%)

Data are shown as median (interquartile range) or number (%)

**Fig. 1:**
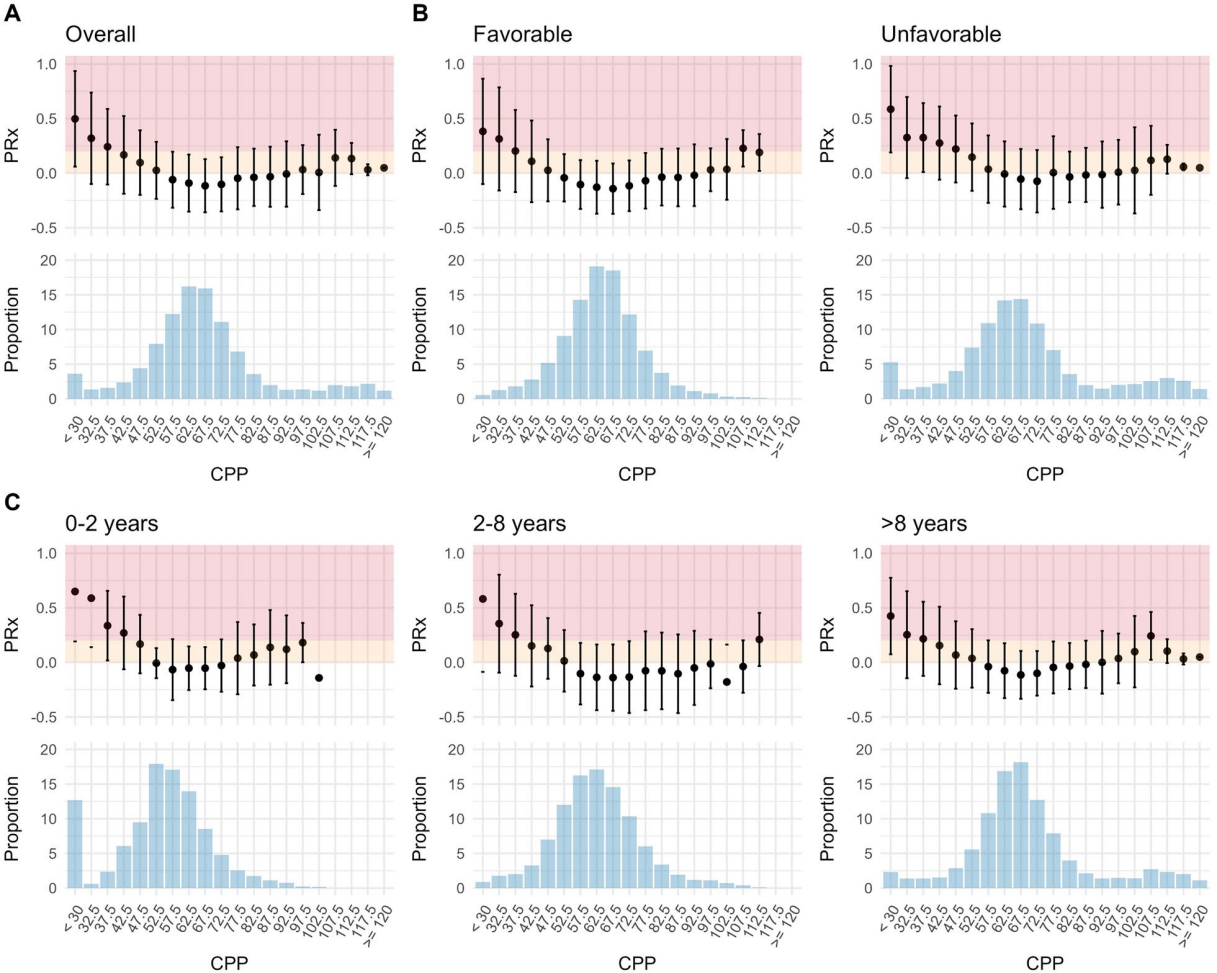
*The relationship between CPP and PRx* The relationship between CPP and the PRx are displayed, considering the full cohort (**a**) or stratified by outcome (**b**) or age (**c**). For all relationships, a clear lower limit can be identified. Conversely, the upper limit seems to be relatively high, occurring largely at CPP levels above 100 mm Hg. CPP cerebral perfusion pressure, PRx pressure reactivity index

Different combinations of CPP and PRx relationships are displayed in Fig. 1. When considering the overall association (Fig. 1a), a distinct linear increase in PRx can be identified when CPP falls below 62.5 mm Hg. Similarly, in the outcome-stratified plots (Fig. 1b), the increase in PRx with CPP below 57.5 mm Hg and 67.5 mm Hg for patients with favorable and unfavorable outcome, respectively, can be identified. The increase in PRx with increasing CPP is very gradual when considering both overall and outcome-stratified plots. PRx appears to only reach 0.2 at CPP levels above 100 mm Hg. Figure 1c displays the age-stratified CPP–PRx relationships. Although PRx reaches 0.2 at CPP of 97.5 mm Hg in patients aged 0–2 years, in the other age groups, PRx reaches 0.2 at CPP of 107.5 and 112.5 mm Hg.

**Fig. 2:**
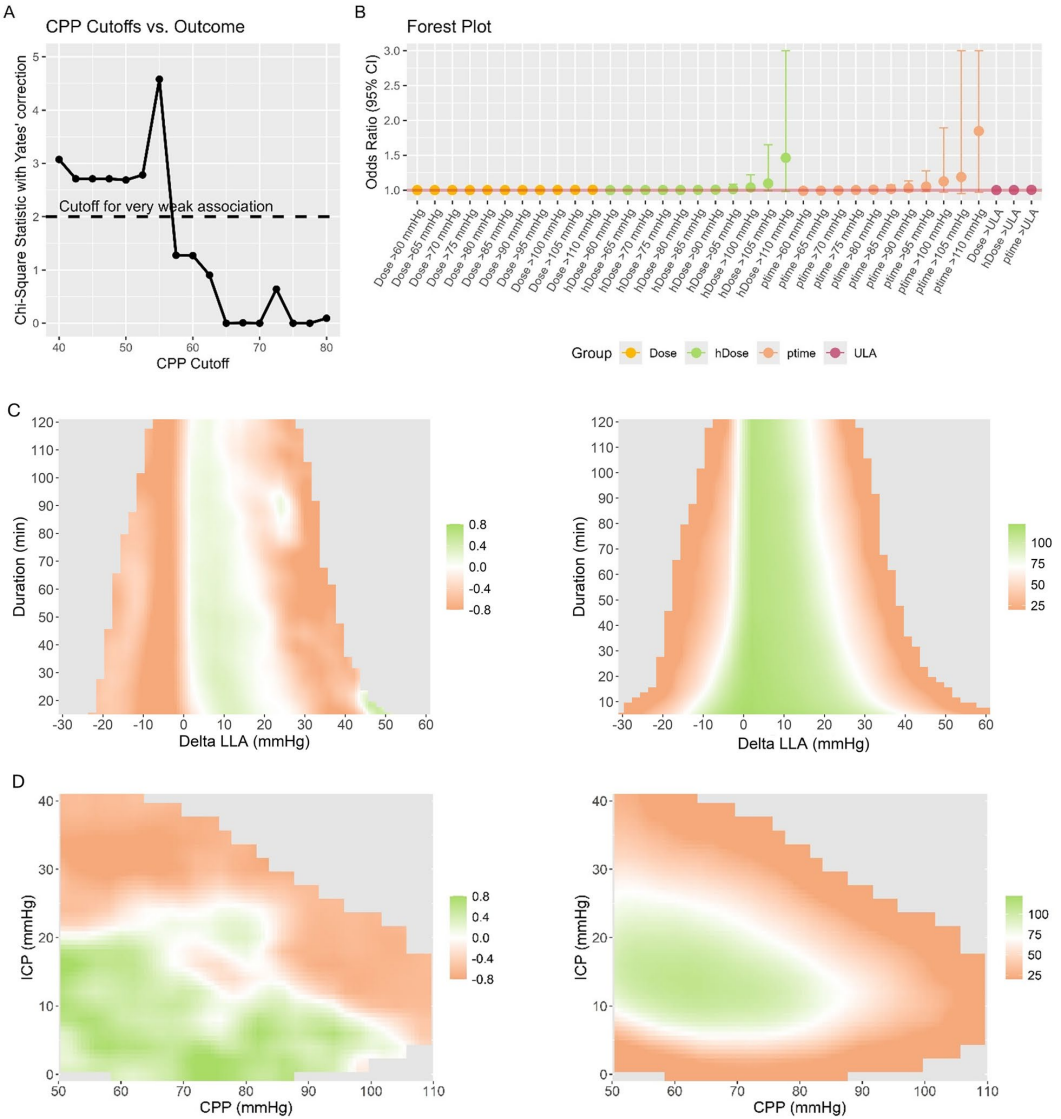
*The association between CPP and outcome* The associations between different levels of CPP and dichotomized outcome are assessed using iterative χ^2^ testing (**a**; considering the overall average CPP) or logistic regression (**b**; considering overall dose, hDose, or ptime relative to the different CPP cutoffs). Panels **c** and **d** display the heatmaps of the association between deviations from the LLA and outcome and associations between different ICP and CPP combinations and outcome, respectively, on the left, and the corresponding data densities (representing the number of patients per bin) are displayed on the right. The results are color coded, with orange representing correlation with unfavorable outcome and green representing correlation with favorable outcome. Overall, a distinct band associated with favorable outcome from CPP directly above the LLA up to around 25 mm Hg above the LLA is identified. Similarly, CPP levels between 50 and 80 mm Hg are associated with favorable outcome as long as ICP remains below 20 mm Hg. For CPP levels above 80 mm Hg, the sensitivity to ICP seems to increase. CPP cerebral perfusion pressure, hDose hourly dose, ICP intracranial pressure, LLA lower limit of autoregulation, ptime percentage time

Figure 2a displays the results of the iterative χ^2^ testing. The critical CPP threshold yielding the highest χ^2^ value for prognostication was identified at 55 mm Hg. Beyond 57.5 mm Hg, no cutoff revealed a significant prognostic value. The results assessing dose, hDose, and ptime instead of overall averages for prognostication are presented in Fig. 2b. Overall, only hDose and ptime displayed a trend toward significance when considering CPP cutoffs beyond 90 mm Hg. Of note, no CPP metric yielded a significant association with outcome (*p* < 0.05). Similarly, even after adjusting the outcome definition using the sliding dichotomy approach, no CPP metric was able to differentiate between patients with favorable and unfavorable outcome (*p* < 0.05). Figure 2c and d illustrate the results of the heatmap analyses. Figure 2c displays the association between deviations from the LLA and outcome. Overall, a distinct band associated with favorable outcome starting from CPP directly above the LLA up to around 25 mm Hg above the LLA could be identified, with a slight decrease when such insults occurred with a duration above one hour. Figure 2d displays the association between different ICP and CPP combinations and outcome. Overall, a relatively consistent association with favorable outcome can be identified when considering the section with ICP below 20 mm Hg and CPP between 50 and 80 mm Hg. All sections with ICP above 20 mm Hg were associated with unfavorable outcome. Additionally, beyond a CPP value of 80 mm Hg (an overall relatively rare occurrence), even lower ICP values displayed an association with worse outcome.

Complications were infrequent, with acute respiratory distress syndrome occurring in three patients and secondary intracranial hemorrhage in five patients. Among these, two patients with acute respiratory distress syndrome and four with secondary hemorrhage achieved a favorable outcome. The median CPP in patients with acute respiratory distress syndrome was 54 mm Hg (IQR 54–70 mm Hg), whereas in those with secondary intracranial hemorrhage, it was 65 mm Hg (IQR 64–67 mm Hg). These CPP values did not significantly differ from those observed in patients without these complications (*p* > 0.5).

## Discussion

We explored, using various methods, the relationship between CPP and outcome after pediatric TBI. Instead of focusing on the established association between low CPP and worse outcome, we assessed the effects of CPP above the suggested limits set by the Brain Trauma Foundation guidelines in the multicenter prospective STARSHIP study. The main findings were as follows:

- Contrasting explorations performed in adults, we were unable to identify a clear upper limit beyond which PRx increases more rapidly (akin to the LLA)
- A trend toward a worse outcome could only be identified when assessing CPP-derived metrics beyond 80–90 mm Hg, a finding that is, however, dependent on the ICP, with higher CPP levels displaying a higher sensitivity to ICP
- Secondary complications (i.e., acute respiratory distress syndrome and secondary intracranial hemorrhage) were infrequent, and no difference in the level of CPP comparing those with and without such secondary complications could be identified

Previous studies have explored the relationship between CPP and PRx following pediatric TBI, highlighting dynamic changes in response to CPP variations [10, 19, 20]. Only two studies have reported a notable increase in PRx with higher CPP values (reflecting CPP closer to the LLA) [19, 20]. In both cases, PRx did not exceed 0.2, leaving relevant uncertainty about the precise upper limits of this relationship. These results contrast with adult explorations, wherein a clearer U-shaped relationship has been identified [21, 22]. In light of recent experimental findings, this result should not be surprising. Klein et al. [23] directly visualized the changes in pial arteriolar blood flow in response to changes in CPP. Interestingly, with CPP increasing beyond the autoregulation range, a multiphasic transition from intact to impaired autoregulation could be seen. They observed first a relatively moderate increase in CBF (corresponding to the inability of small arterioles to constrict any further), followed by a progressively steeper increase (reflecting passive reactivity, which occurred after CPP completely overpowered the arteriolar myocyte constriction of arterioles of all sizes). In contrast, passive reactivity below the LLA reflects a passive effect because even maximal dilation does not allow for further decrease in cerebrovascular resistance [24]. Although the magnitude of decrease in cerebrovascular resistance by vasodilation to counteract decreases in CPP largely depends on the maximal diameter that can be reached, the magnitude of vasoconstriction to counteract increases in CPP depends on the maximal tone that the smooth muscle cells can exert. This maximal tone depends on different physiological factors that affect their function (e.g., arteriosclerosis with thickening of vessel wall, fibrosis, and inflammation, which is a common consequence of chronic hypertension and type 2 diabetes) and decreases with age [25–27]. These age-dependent differences might explain why pediatric cohorts portray a lower vulnerability to high CPP.

The results are derived from a pediatric cohort of 135 patients. As an observational trial, this study did not set out to assess the safety of any CPP cutoffs. Consequently, we can only report on associations rather than prove the presence (or lack of) causality between the physiological findings and outcome. Overall, patients with favorable outcome generally have higher CPP during their intensive care unit stay [28, 29]. There exist some preliminary findings in adult TBI [8] describing the association between higher CPP targets and the occurrence of acute respiratory distress syndrome, albeit without significantly affecting outcomes. In the pediatric TBI cohorts, such associations have not been explored in depth yet. Overall, the incidence of acute respiratory distress syndrome is substantially lower in pediatric intensive care unit cohorts compared to adult patients [30, 31]. Even so, hypertension (defined as readings above the standardized 99th percentile) is certainly associated with secondary complications, such as acute kidney injury [32]. Although the overall incidence of complications in our cohort was low, we acknowledge that our focus on secondary hemorrhage and acute respiratory distress syndrome likely underestimates the broader spectrum of potential secondary injuries associated with elevated CPP. Subclinical manifestations, such as hyperemic edema, neuroinflammation, or regional blood–brain barrier disruption, may occur in the absence of overt systemic complications yet may still meaningfully affect outcome. These consequences were not assessed as part of the original study protocol [12] and therefore remain a limitation of this analysis. Similarly, there are many differences in physiology that arise depending on the age of the patients or the phenotype of the injury. However, the available number of patients is insufficient for further age-based or disease characteristic–based stratification [12].

The current study also highlights some shortcomings of the currently used CPPopt algorithm. In line with experimental data [23] and previous explorations [10, 19, 20], we found an asymmetry in the relationship between CPP and PRx, with a steeper increase below the LLA and a relatively moderately slow increase when CPP increased toward the ULA. The current algorithm [14] expects a bilaterally symmetric U-shaped curve. Consequently, the full curve is fit even if the upper limit is not reached. This approach is likely inadequate in the pediatric patient cohort, in which there is a distinct asymmetry, with a wide autoregulation plateau. This is to say that because PRx remains low across a broad range of CPP values, this results in a wide autoregulatory plateau. The algorithm is prone to fitting variable CPPopt locations across this range rather than precisely identifying one value because, in these cases, the CPPopt is reflected by a range rather than a single CPP value. In contrast, the LLA is more reliably estimated based on the linear rise in PRx beyond a value of 0.2 as CPP decreases. Because of this limitation, we did not perform outcome analyses based on thresholds above/below the CPPopt, as this could have introduced significant estimation bias. This limitation should be considered when interpreting studies that rely on the CPPopt in pediatric TBI, and future efforts should aim to develop improved algorithms that account for the asymmetry and extended plateau observed in this population. Lastly, our data do not exclude the possibility that very high CPP may negatively influence outcome; however, they emphasize that if such an effect exists, it is considerably less pronounced than the negative impact associated with low CPP [33]. Along similar lines, PRx represents a global surrogate of cerebrovascular autoregulation and may not reflect regional variations in autoregulatory capacity. It does not necessarily correlate with more localized measures derived from modalities such as CBF velocity or near-infrared spectroscopy [34]. As such, it is important to acknowledge that specific brain regions may still experience hyperemia, hypoperfusion, or pressure-passive flow, even when global PRx values suggest preserved autoregulation [2].

## Conclusions

Pediatric patients appear to be relatively resilient to CPP values beyond those suggested by the Brain Trauma Foundation guidelines, with only values exceeding 80–90 mm Hg showing a trend toward worse outcomes. Similarly, no difference in CPP depending on the incidence of complications could be identified. Considering the established association between low CPP and poor outcomes, our findings suggest that exposure to higher absolute CPP values may not be associated with worse outcomes in this cohort. However, this study cannot prove the safety of high CPP values, particularly across different age groups. Given the observational nature of the study and the potential for confounding factors, including the means by which higher CPP was achieved, these results should be interpreted with caution. Rather than supporting a definitive clinical recommendation, our findings underscore the need for prospective trials to evaluate the safety and efficacy of targeting higher absolute CPP values in pediatric TBI.

## Supplementary Information

Below is the link to the electronic supplementary material.

Supplementary file1 (PDF 76 kb)

## Data Availability

Postprocessed data are available upon reasonable request to the corresponding author.
